# Study on the effective dose of alfentanil combined with ciprofol in inhibiting intraoperative body movement responses in patients undergoing breast lump resection

**DOI:** 10.3389/fmed.2025.1720728

**Published:** 2026-01-07

**Authors:** Fei Li, Yingying Lu, Gang Lu, Jiaqi Gu, Yumin Zhu

**Affiliations:** 1 Department of Anesthesiology, The Affiliated Suzhou Hospital of Nanjing Medical University, Suzhou Municipal Hospital, Gusu School, Nanjing Medical University, Suzhou, China; 2 Department of Anesthesiology, The First Affiliated Hospital of Nanjing Medical University, Nanjing, Jiangsu, China

**Keywords:** alfentanil, ED_50_, ED_95_, intravenous anesthesia, segmental mastectomy

## Abstract

**Background:**

Alfentanil and ciprofol are used in combination for intravenous anesthesia, but the effective dose of alfentanil required to suppress movement during surgery when co-administered with ciprofol is not well-defined. This study aimed to determine the effective dose of alfentanil, combined with ciprofol, required to inhibit body movement responses in patients undergoing breast lumpectomy.

**Methods:**

Patients scheduled for elective breast lumpectomy uswere selected. Ciprofol was administered intravenously at anesthesia induction, and alfentanil was given using the sequential method with an initial dose of 10 μg/kg and a dose gradient of 1 μg/kg. The dosage of alfentanil, the incidence of intraoperative respiratory depression, hypotension requiring treatment, and bradycardia were recorded. Postoperative adverse reactions were assessed and recorded. Probit regression analysis was used to calculate the effective dose.

**Results:**

The median effective dose (ED₅₀) of alfentanil for inhibiting intraoperative body movement was calculated to be 13.396 μg/kg, with a corresponding 95% effective dose (ED_95_) of 15.752 μg/kg. During the surgical procedure, no patients required therapeutic intervention for either intraoperative hypotension or bradycardia. However, three patients developed respiratory depression. Notably, no postoperative adverse reactions were documented in any of the study participants.

**Conclusion:**

The ED_50_ of alfentanil combined with ciprofol for intravenous anesthesia to suppress movement during breast lumpectomy is 13.396 μg/kg, and the ED₉₅ is 15.752 μg/kg.

**Clinical trial registration:**

http://www.chictr.org.cn, ChiCTR2500109829.

## 1 Introduction

Elective breast lumpectomy is minimally invasive with short operative times. Intravenous anesthesia provides rapid onset and prompt postoperative recovery, thereby not only fulfilling surgical needs but also enhancing patient comfort and lowering the economic burden on patients (1, 2). Alfentanil, as a preferred medication for short perioperative procedures, offers advantages such as rapid onset, good controllability, and quick recovery. It is currently widely used in clinical practice for minor surgeries like painless endoscopy examination (3–5). Ciprofol is a new short-acting intravenous anesthetic with a chemical structure similar to propofol but offers more stable hemodynamics and less injection pain (6–8). Alfentanil Hydrochloride Injection produced by Yichang Humanwell Pharmaceutical Co., Ltd. and Cipepofol Injection manufactured by Shenyang Haisco Pharmaceutical Co., Ltd. are both registered with the National Medical Products Administration (NMPA), with approval numbers H20203054 and H20200013, respectively. The combined use of alfentanil and ciprofol in breast lumpectomy may reduce the required dose of each single drug, decrease postoperative adverse reactions, and achieve rapid induction and smooth recovery (9). However, the specific dose–response relationship has not been clearly defined, and the balance between inhibiting intraoperative body movement responses and maintaining respiratory and circulatory stability needs exploration. This study aimed to determine the median effective dose (ED_50_) and 95% effective dose (ED_95_) of alfentanil combined with ciprofol for intravenous anesthesia in inhibiting body movement responses during breast lumpectomy.

## 2 Materials and methods

### 2.1 Study subjects

The CONSORT guidelines were followed (see the CONSORT Checklist in the Supplemental material) (10). The study protocol was approved by the Ethics Committee of Suzhou Municipal Hospital (Approval No. K-2025-118-K01). Written informed consent was obtained from all participants after the study procedures had been fully explained by the investigators. The study was conducted in accordance with the Declaration of Helsinki. Patients scheduled for breast lumpectomy under intravenous anesthesia at Suzhou Municipal Hospital were selected. Inclusion criteria: Female patients aged 18–65 years; Body Mass Index (BMI) 18.5–25.0 kg/m^2^; American Society of Anesthesiologists (ASA) I–II; breast lump size <5 cm. Exclusion criteria: Difficult airway; long-term preoperative use of opioids or sedatives; long-term alcohol use; long-term smoking; recent history of asthma attack or upper respiratory tract infection; allergy to anesthetic drugs. This study was approved by the hospital’s ethics committee, and all patients provided written informed consent.

### 2.2 Anesthesia induction and management

Patients fasted routinely for 8 h and abstained from fluids for 2 h preoperatively, with no pre-anesthetic medication. Upon entering the operating room, intravenous access was established, oxygen was administered via nasal cannula at 2–5 L/min, and blood pressure (BP), pulse oxygen saturation (SpO₂), and heart rate (HR) were routinely monitored. Vital signs were recorded once the patient was calm. After anesthesia initiation, ciprofol (Shenyang Haisco Pharmaceutical Co., Ltd., China. Indications: This agent is designated for: Sedation and anesthesia for procedures not requiring tracheal intubation; Induction and maintenance of general anesthesia; Sedation during mechanical ventilation in critical care settings) was injected intravenously at 0.5 mg/kg, followed by a continuous infusion at 0.6 mg/kg/h. Alfentanil (Yichang Humanwell Pharmaceutical Co., Ltd., China. Indications: Alfentanil is indicated as an anesthetic analgesic for the induction and maintenance of general anesthesia) was slowly injected intravenously. Surgery could only begin when the eyelash reflex disappeared and the Modified Observer’s Assessment of Alertness/Sedation (MOAA/S) score was ≤1 (11, 12). All surgeries were performed by the same experienced breast surgeon, and anesthesia was administered by the same anesthesiologist. Intraoperative involuntary body movement that interfered with the surgeon’s operation was considered a positive body movement response, prompting administration of additional alfentanil. If intraoperative SpO₂ fell below 90%, oxygen flow was increased and the jaw was lifted. If SpO₂ decreased further, mask ventilation with positive pressure was performed, and tracheal intubation was done if necessary. Ephedrine (6 mg) was administered intravenously if the mean arterial pressure (MAP) decreased by more than 20% from baseline or fell below 65 mmHg. If the HR was less than 50 bpm, atropine 0.5 mg was administered intravenously (12).

### 2.3 Sequential method

The modified Dixon’s up-and-down sequential method was used for alfentanil administration. This study was a dose-finding study designed as a sequential experiment. Based on previous studies, the sample size was approximately 40 cases, and no exact sample size calculation was performed. According to pre-experimental results and relevant studies, the initial intravenous dose of alfentanil for the first patient was set at 10 μg/kg, with a dose step of 1 μg/kg between consecutive patients. If the previous patient had a positive body movement response, the alfentanil dose for the next patient was increased by one step. If no body movement response interfering with surgery occurred during the procedure, it was judged as a negative response, and the alfentanil dose for the next patient was decreased by one step. The study was terminated when 7 crossover points (from negative to positive responses) occurred (13).

### 2.4 Observation indicators

The dosage of alfentanil used was recorded. MAP, HR, and SpO₂ were recorded before anesthesia induction, after anesthesia induction, after surgery began, and upon awakening. The occurrence of adverse reactions such as hypotension, bradycardia, hypoxemia, postoperative nausea and vomiting, and emergence agitation were observed and recorded.

### 2.5 Statistical processing

SPSS 25.0 was used for data analysis, and GraphPad Prism 9 for graphing. Measurement data for vital signs conforming to a normal distribution are expressed as mean ± standard deviation (*x̄* ± *s*). The baseline characteristics between groups were compared using independent-samples *t*-tests. The differences in hemodynamic parameters over time were analyzed using a one-way repeated-measures ANOVA. *p* < 0.05 was considered statistically significant. Probit regression analysis was used to calculate the ED₅₀ and ED₉₅ of alfentanil.

## 3 Results

**Figure 1:**
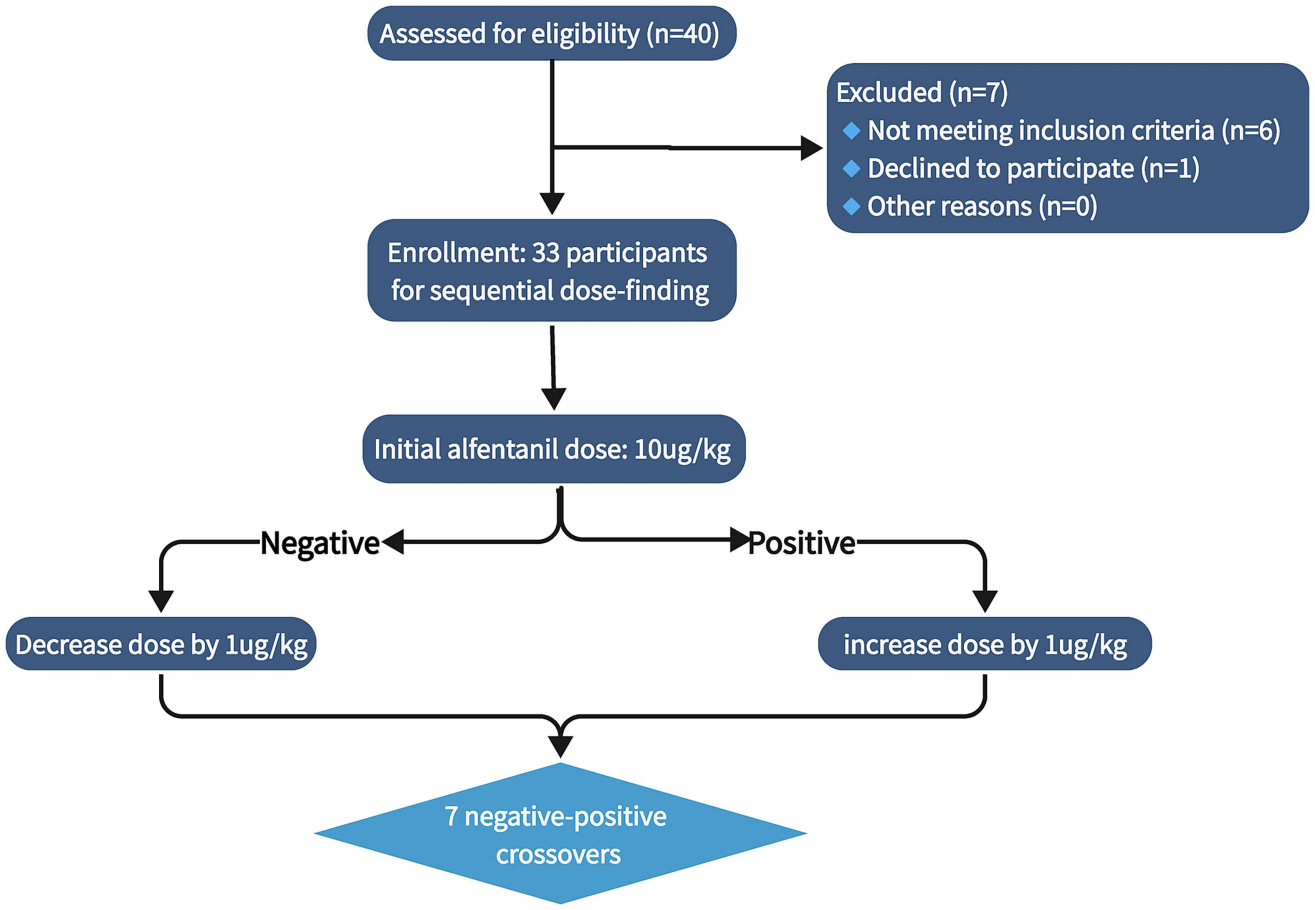
Flow diagram of the study.

A total of 40 patients were initially enrolled in the study from August, 2025, to October, 2025. After excluding 7 individuals who did not meet the inclusion criteria or declined to participate, 33 patients were finally included for dose exploration (Figure 1). All participants were of Asian descent. The general demographic and clinical characteristics of the patients are presented in Table 1. There were no significant differences in demographic data (age, weight, height, BMI) or clinical characteristics between the positive and negative groups. Aside from hypertension (3 patients in the positive group and 1 in the negative group), no other comorbidities were documented in either group. All patients successfully completed the surgery without circulatory adverse events such as hypotension or bradycardia. Postoperative recovery was smooth, and no common anesthesia complications such as postoperative nausea and vomiting or emergence agitation were observed.

**Table 1:** Demographic characteristics of the subjects.

Items	Positive group (*n* = 18)	Negative group (*n* = 15)	*P*
Age, years	37.06 ± 11.91	42.13 ± 11.75	0.229
Height, cm	162.56 ± 4.06	160.20 ± 3.63	0.092
Weight, kg	51.56 ± 5.69	55.93 ± 3.15	0.984
BMI, kg/m^2^	21.80 ± 2.15	21.80 ± 1.11	0.996
ASA status			
I, n	10	8	
II, n	8	7	
Hypertension, n	3	1	
Other comorbidity, n	0	0	

BMI, body mass index; ASA, American Society of Anesthesiologists.

During anesthesia induction, 3 patients experienced transient respiratory depression (respiratory rate <8 breaths/min or SpO₂ < 90%). After jaw thrust maneuver, assisted ventilation, and increased oxygen flow (6 L/min), SpO₂ quickly returned to the normal range (≥98%) without affecting the surgical procedure. Hemodynamic and oxygenation parameters are summarized in Table 2. A repeated-measures analysis of variance revealed a significant main effect of time on MAP, HR, and SpO₂ (all *p* < 0.05). MAP was significantly lower at post-induction (T1), surgical incision (T2), and emergence (T3) compared to the pre-anesthesia baseline (T0) (all *p* < 0.001), with mean differences (MD) and 95% confidence intervals (CI) as follows: T1 vs. T0 (MD = 7.97 mmHg, 95% CI: 5.17 to 10.77, *p* < 0.001); T2 vs. T0 (MD = 11.52 mmHg, 95% CI: 9.22 to 13.82, *p* < 0.001); T3 vs. T0 (MD = 5.21 mmHg, 95% CI: 2.98 to 7.44, *p* < 0.001). MAP reached its nadir at T2 (80.97 ± 9.62 mmHg), which was significantly lower than that at T1 (MD = 3.55 mmHg, 95% CI: 0.78 to 6.31, *p* = 0.014). At emergence (T3), MAP increased significantly compared to T2 (MD = −6.30 mmHg, 95% CI: −8.11 to −4.49, *p* < 0.001) but remained significantly lower than the baseline (*p* < 0.001). A comparable reduction was observed in HR. Significant decreases were found at T1 and T2 compared to T0 (both *p* < 0.001): T1 vs. T0 (MD = 9.36 bpm, 95% CI: 6.18 to 12.55, *p* < 0.001); T2 vs. T0 (MD = 10.88 bpm, 95% CI: 7.03 to 14.73, *p* < 0.001). The lowest HR value was also recorded at T2 (63.21 ± 8.59 bpm). By T3, HR had recovered to a level (72.79 ± 8.14 bpm) that was not significantly different from the baseline (74.09 ± 10.96 bpm), but it was significantly higher than the values at both T1 and T2 (both *p* < 0.001). For SpO₂, a transient but significant decrease was observed only at T1 (96.67 ± 2.70%) compared to T0 (MD = 2.00, 95% CI: 0.68 to 1.75, *p* < 0.001). SpO₂ subsequently returned to levels comparable to baseline at both T2 and T3, with no significant difference versus T0, but were significantly higher than the value at T1 (T2 vs. T1: MD = 1.69, 95% CI: 0.34 to 1.48, *p* = 0.003; T3 vs. T1: MD = 1.42, 95% CI: 0.04 to 1.24, *p* = 0.038). In conclusion, the combination of ciprofol and alfentanil for anesthesia in patients undergoing lumpectomy resulted in an expected reduction in MAP and HR during induction and early surgery, with all values remaining within acceptable clinical limits and demonstrating rapid recovery by emergence. The effect on SpO₂ was minimal and transient, indicating a favorable hemodynamic stability and respiratory safety profile for this anesthetic regimen.

**Table 2:** Comparison of MAP, HR and SpO_2_ at different time points (*n* = 33).

Items	T0	T1	T2	T3
MAP (mmHg)	92.48 ± 11.51	84.52 ± 12.54*	80.97 ± 9.62*^a^	87.27 ± 7.88*^b^
HR (bpm)	74.09 ± 10.96	64.73 ± 8.06*	63.21 ± 8.59*	72.79 ± 8.14^ab^
SpO_2_ (%)	98.67 ± 1.22	96.67 ± 2.70*	98.36 ± 1.27^a^	98.09 ± 1.13^a^

Data are presented as mean ± standard deviation. T0: pre-anesthesia; T1: post-induction; T2: surgical incision; T3: emergence.

MAP, mean arterial pressure; HR, heart rate; SpO_2_, pulse oximetry saturation.

*Compared with pre-anesthesia (T0), *p* < 0.05. ^a^Compared with post-induction (T1), *p* < 0.05. ^b^Compared with surgical incision (T2), *p* < 0.05.

**Figure 2:**
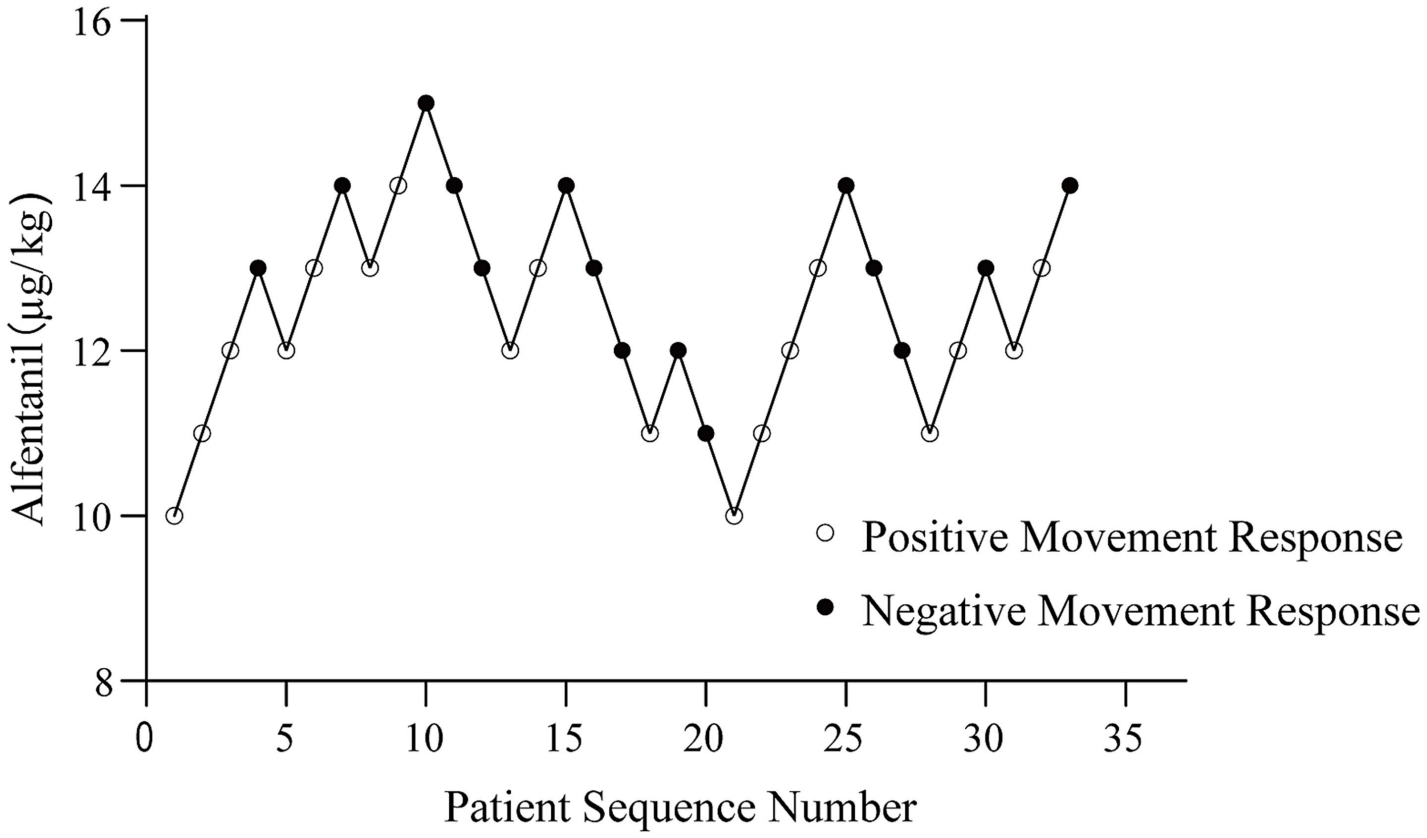
Sequential trial diagram of alfentanil combined with ciprofol in suppressing patient movement response during lumpectomy.

A sequential trial (up-down) design was employed to determine the effective dose of alfentanil combined with ciprofol for inhibiting body movement during breast lumpectomy. The trial, which was terminated upon the occurrence of seven negative–positive crossovers, enrolled 33 patients. Within the tested alfentanil dose range of 10–15 μg/kg, body movement responses were observed in 18 patients (positive) and absent in 15 (negative). A dose-effect relationship was established by Probit regression, yielding the equation *p* = −9.351 + 0.698 × dose (goodness-of-fit *p* = 0.870), indicating a well-fitted model. The sequential trial results are illustrated in Figure 2.

**Figure 3:**
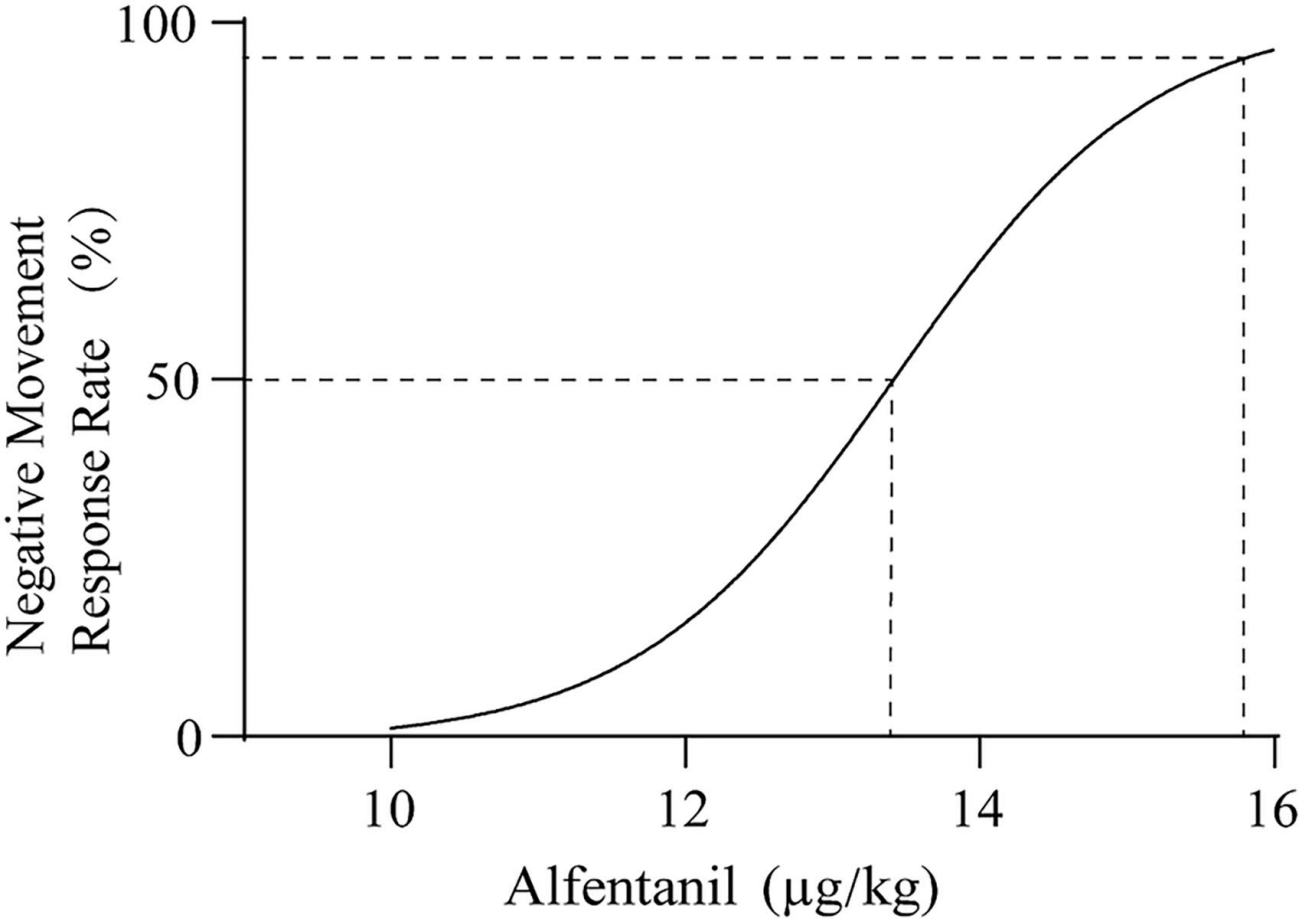
Dose-effect curve of alfentanil combined with ciprofol in suppressing patient movement response during lumpectomy.

Further analysis showed that when combined with ciprofol, the ED₅₀ of alfentanil for inhibiting body movement responses during breast lumpectomy was 13.396 μg/kg (95% CI: 12.803–14.016 μg/kg), and the ED₉₅ was 15.752 μg/kg (95% CI: 14.922–17.423 μg/kg). Figure 3 shows the sigmoidal curve relationship between the alfentanil dose and the probability of movement inhibition. The slope of the curve indicates a clear dose-dependent effect of alfentanil on movement inhibition within the 13–16 μg/kg dose range.

## 4 Discussion

Breast lumpectomy, as a common treatment for benign breast lesions, offers minimally invasive advantages such as minimal trauma and quick recovery (14, 15). However, during the procedure, mechanical and thermal insults such as tissue traction and heat stimulation from high-frequency electrocautery can still trigger involuntary body movement responses in patients. These nociceptive stimuli are transmitted via peripheral nerves to the central nervous system. Without intervention, this can lead to intraoperative movement and hypertensive stress responses. To effectively inhibit such nociceptive transmission, high doses of opioids (such as remifentanil, sufentanil) are often required clinically to maintain adequate analgesic depth (16, 17). Compared to traditional opioids, the rapid onset and short duration of action of alfentanil make it more suitable for short procedures or operations requiring fine control of analgesic depth (18).

Alfentanil is an ultra-short-acting μ-opioid receptor agonist. By selectively acting on the μ-receptors in the central nervous system, it effectively inhibits the transmission of pain signals to higher centers, providing rapid and precise analgesia. Its pharmacokinetic properties are excellent, with a distribution half-life of only about 1.5 min, allowing rapid achievement of effective blood concentration; the elimination half-life is about 90 min, resulting in lower risk of drug accumulation and more controllable postoperative awakening time (19). Ciprofol is a new intravenous sedative whose mechanism of action primarily relies on enhancing the activity of γ-aminobutyric acid (GABA) receptors, thereby producing stable sedation. Compared to propofol, ciprofol causes slower opening of lipid membrane channels and reduces histamine release, diminishing its inhibitory effect on the cardiovascular system and resulting in more stable hemodynamics (8, 20). Furthermore, the metabolism of ciprofol does not depend on the liver and kidneys, giving it an advantage in special patient populations (e.g., those with hepatic or renal insufficiency) (21).

The combination of alfentanil and ciprofol can produce a synergistic effect, significantly reducing the required dose of each single drug and lowering the incidence of adverse reactions (22). The potent analgesic effect of alfentanil compensates for the lack of analgesia from ciprofol, while the stable sedation provided by ciprofol reduces the risk of respiratory depression caused by opioids (23). Their combination thus facilitates a more rapid and profound sedative-analgesic effect. This synergy is demonstrated by a significant reduction in anesthesia induction time (2.51 ± 1.0 min (8) for the combination vs. 12.58 ± 0.7 min (24) for ciprofol alone). Furthermore, the addition of alfentanil reduced the required dose of ciprofol for achieving the same induction endpoint from 0.5 mg/kg to 0.3 mg/kg (8, 25). The results indicated that the combination regimen was associated with a lower incidence of intervention-requiring respiratory depression and no episodes of hypotension or bradycardia requiring treatment, demonstrating a clear advantage over comparable propofol-alfentanil regimens. The absence of postoperative nausea and vomiting may be attributable to alfentanil’s high selectivity for *μ*-receptors, along with its weaker activity at δ- and κ-receptors, which collectively help mitigate gastrointestinal side effects (26, 27). Compared to propofol, the hemodynamic stability of ciprofol was further highlighted in this study: the induction dose of propofol alone often requires 1.5–2.5 mg/kg, and the risk of hypotension increases when combined with opioids. In contrast, this protocol used ciprofol at 0.5 mg/kg for induction combined with 0.6 mg/kg/h for maintenance, potentially reducing circulatory fluctuations through smoother blood concentration changes. Additionally, the short-acting nature of alfentanil contributes to rapid postoperative awakening, following a similar logic to gastrointestinal endoscopy anesthesia management strategies combining remimazolam (28).

While the potential pharmacokinetic interactions between ciprofol and alfentanil remain unexplored *in vivo*, it is reasonable to hypothesize that their interaction may mirror that of propofol and alfentanil, given that ciprofol shares an identical mechanism of action with propofol. The well-documented interaction between propofol and alfentanil provides a valuable reference. The locus coeruleus, which contains receptors for both GABA and opioids, is implicated as a key site for their actions and interactions (29). Alfentanil increases the central compartment volume of distribution and clearance of propofol, leading to an approximately 20% elevation in propofol blood concentrations when co-administered (30). This pharmacokinetic interaction may be mediated by opioid-induced alterations in hemodynamics (e.g., cardiac output, hepatic blood flow) or tissue distribution. Furthermore, concomitant administration with propofol results in elevated plasma concentrations of alfentanil (30). One proposed mechanism is that propofol may inhibit cytochrome P450 (CYP450) enzymes *in vitro*, thereby attenuating the oxidative metabolism of alfentanil. However, this mechanism is primarily based on *in vitro* studies, and its clinical significance in humans requires further validation.

This study, through dose-effect analysis, confirmed that the ED₅₀ of alfentanil combined with ciprofol for inhibiting body movement responses during breast lumpectomy is 13.396 μg/kg, and the ED₉₅ is 15.752 μg/kg. This dose range is significantly lower than traditional opioid regimens, reflecting the synergistic effect of the two drugs. It is worth noting that the ED₉₅ value obtained in this study (15.752 μg/kg) is close to the highest dose set in the trial (15 μg/kg), suggesting that individualized dose adjustment strategies may need to be considered in clinical practice, especially for patients with stronger surgical stimulation or lower sensitivity to opioids. Simultaneously, this dose range is lower compared to the effective dose of alfentanil used alone, confirming a synergistic effect between ciprofol and alfentanil. Clinical observations showed that this combined anesthesia regimen not only effectively inhibited body movement responses caused by surgical stimulation but also maintained good hemodynamic stability, with intraoperative fluctuations in MAP and HR controlled within 20% of baseline values. Particularly noteworthy is that only 3 patients (9.1%) experienced transient respiratory depression, which was quickly resolved with simple interventions without affecting the surgical procedure.

The advantage of this anesthesia regimen lies in its precise alignment with the core requirements of day surgery: “short-acting, controllable, safe.” The rapid onset and short duration of alfentanil (distribution half-life 1.5 min) ensure precise control of anesthetic depth, while the stable hemodynamic properties and low histamine release characteristics of ciprofol reduce the risk of circulatory fluctuations. The combination provides adequate intraoperative analgesia while facilitating rapid postoperative awakening, aligning with the Enhanced Recovery After Surgery (ERAS) principle of “precision anesthesia and enhanced recovery.”

This study has certain limitations: (1) Although the sequential method saves sample size, 33 patients may affect the precision of the ED₅₀/ED₉₅ confidence intervals. Future multi-center, large-sample clinical trials are needed to further validate the universality of this dosing regimen and refine the dose–response curves for different populations (e.g., elderly, obese patients). (2) Intraoperative body movement responses relied on the surgeon’s subjective judgment and were not combined with objective indicators (such as BIS monitoring or electromyography), which may introduce bias. (3) Fixed ciprofol dose: Ciprofol was administered at a fixed infusion dose; the dose interaction between ciprofol and alfentanil was not explored. Future studies could design response surface models to optimize the ratio. (4) Lack of long-term data: Postoperative 24-h pain scores or patient satisfaction were not assessed, potentially underestimating the residual analgesic effect of alfentanil.

Future research needs to further explore the dose adaptability of alfentanil-ciprofol in different types of surgery and optimize infusion strategies combined with pharmacokinetic models. Simultaneously, it is worthwhile to explore synergistic strategies between this regimen and multimodal analgesia, such as regional blocks (e.g., intercostal nerve block) and NSAIDs, aiming to further reduce opioid usage while optimizing postoperative analgesia and reducing adverse reactions, providing better solutions for day surgery anesthesia. Furthermore, personalizing differences through genetic polymorphism analysis and adjusting doses based on patient weight, ASA status, and pain sensitivity, especially for the elderly or those with hepatic/renal insufficiency, may achieve precise anesthesia. Establishing population pharmacokinetic/pharmacodynamic models for alfentanil-ciprofol could enable precise administration via target-controlled infusion.

## 5 Conclusion

This study showed that the ED₅₀ of alfentanil combined with ciprofol for inhibiting body movement responses during breast lumpectomy is 13.396 μg/kg, and the ED₉₅ is 15.752 μg/kg. This regimen ensures anesthetic effectiveness while demonstrating good hemodynamic stability and a low risk of respiratory depression. Future large-sample studies are needed to refine the dose–response curve and explore synergistic strategies with multimodal analgesia.

## Data Availability

The original contributions presented in the study are included in the article/supplementary material, further inquiries can be directed to the corresponding authors.

## References

[1] Agarwal G Sattavan S Vishvak Chanthar KMM Kumar A Sabaretnam M Chand G . Cost-efficacy analysis of use of frozen section histology for margin assessment during breast conservation surgery in breast cancer patients. World J Surg. (2023) 47:2457–63. doi: 10.1007/s00268-023-07094-2, 37386245

[2] Boughey JC Hieken TJ Jakub JW Degnim AC Grant CS Farley DR . Impact of analysis of frozen-section margin on reoperation rates in women undergoing lumpectomy for breast cancer: evaluation of the National Surgical Quality Improvement Program data. Surgery. (2014) 156:190–7. doi: 10.1016/j.surg.2014.03.025, 24929768

[3] Lei X Zhang T Huang X. Comparison of a single intravenous infusion of alfentanil or sufentanil combined with target-controlled infusion of propofol for daytime hysteroscopy: a randomized clinical trial. Ther Adv Drug Saf. (2024) 15:20420986241292231. doi: 10.1177/20420986241292231, 39493926 PMC11528634

[4] Shi W Cheng Y He H Fang Q Hu Y Xu X . Efficacy and safety of the remimazolam-alfentanil combination for sedation during gastroscopy: a randomized, double-blind, single-center controlled trial. Clin Ther. (2022) 44:1506–18. doi: 10.1016/j.clinthera.2022.09.014, 36763995

[5] Wang L Wu Q Wang M Ming W Sheng C Zhang Y . The safety and efficacy of alfentanil combined with midazolam in fiberoptic bronchoscopy sedation: a randomized, double-blind, controlled trial. Front Pharmacol. (2022) 13:1036840. doi: 10.3389/fphar.2022.1036840, 36339547 PMC9634630

[6] Akhtar SMM Fareed A Ali M Khan MS Ali A Mumtaz M . Efficacy and safety of Ciprofol compared with Propofol during general anesthesia induction: a systematic review and meta-analysis of randomized controlled trials (RCT). J Clin Anesth. (2024) 94:111425. doi: 10.1016/j.jclinane.2024.111425, 38412619

[7] Cheng X Zhang P Jiang D Fang B Chen F. Safety and efficacy of ciprofol versus propofol for gastrointestinal endoscopy: a meta-analysis. BMC Gastroenterol. (2025) 25:130. doi: 10.1186/s12876-025-03734-0, 40033212 PMC11877735

[8] Zhang J Liu R Bi R Li X Xu M Li L . Comparison of ciprofol-alfentanil and propofol-alfentanil sedation during bidirectional endoscopy: a prospective, double-blind, randomised, controlled trial. Dig Liver Dis. (2024) 56:663–71. doi: 10.1016/j.dld.2023.09.016, 37813808

[9] Gao R Li SX Zhou YH Xing L Fu JP Shen JJ . The median effective dose (ED(50)) and the 95% effective dose (ED(95)) of alfentanil in inhibiting responses to cervical dilation when combined with ciprofol during hysteroscopic procedure: a prospective, double-blind, dose-finding clinical study. BMC Anesthesiol. (2025) 25:353. doi: 10.1186/s12871-025-03217-5, 40684098 PMC12275378

[10] Schulz KF Altman DG Moher D. CONSORT 2010 statement: updated guidelines for reporting parallel group randomised trials. BMJ. (2010) 340:c332. doi: 10.1136/bmj.c332, 20332509 PMC2844940

[11] Chon JY Seo KH Lee J Lee S. Target-controlled infusion of remimazolam effect-site concentration for total intravenous anesthesia in patients undergoing minimal invasive surgeries. Front Med (Lausanne). (2024) 11:1364357. doi: 10.3389/fmed.2024.1364357, 38695029 PMC11061366

[12] Lu Z Zhou N Li Y Yang L Hao W. Up-down determination of the 90% effective dose (ED90) of remimazolam besylate for anesthesia induction. Ann Palliat Med. (2022) 11:568–73. doi: 10.21037/apm-22-89, 35249335

[13] Gui YK Zeng XH Xiao R Xi WF Zhang D Liu Y . The effect of Dezocine on the median effective dose of Sufentanil-induced respiratory depression in patients undergoing spinal anesthesia combined with low-dose Dexmedetomidine. Drug Des Devel Ther. (2023) 17:3687–96. doi: 10.2147/DDDT.S429752, 38090026 PMC10712329

[14] Weaver M Stuckey A. Benign Breast Disorders. Obstet Gynecol Clin N Am. (2022) 49:57–72. doi: 10.1016/j.ogc.2021.11.003, 35168773

[15] Kong X Chen X Jiang L Ma T Han B Yang Q. Periareolar incision for the management of benign breast tumors. Oncol Lett. (2016) 12:3259–63. doi: 10.3892/ol.2016.5117, 27899991 PMC5103926

[16] Li N Qi X Bao J Gu Y Zhou X Wang T . A comparative study of Esketamine-Propofol and Sufentanil-Propofol for analgesia and sedation during breast minimally invasive rotary resection with local anesthesia: a randomized double-blind clinical trial. Drug Des Devel Ther. (2024) 18:5397–407. doi: 10.2147/DDDT.S487872, 39618428 PMC11606144

[17] Fei X Song R Yu X Zhang S Zhang Y Gao Y . The clinical significance of complete process management for the quality control of horizontal rotational resection of a breast mass. Heliyon. (2023) 9:e13537. doi: 10.1016/j.heliyon.2023.e13537, 36865481 PMC9970895

[18] Dong SA Guo Y Liu SS Wu LL Wu LN Song K . A randomized, controlled clinical trial comparing remimazolam to propofol when combined with alfentanil for sedation during ERCP procedures. J Clin Anesth. (2023) 86:111077. doi: 10.1016/j.jclinane.2023.111077, 36764022

[19] Hughes LM Irwin MG Nestor CC. Alternatives to remifentanil for the analgesic component of total intravenous anaesthesia: a narrative review. Anaesthesia. (2023) 78:620–5. doi: 10.1111/anae.15952, 36562193

[20] Liu Y Peng Z Liu S Yu X Zhu D Zhang L . Efficacy and safety of ciprofol sedation in ICU patients undergoing mechanical ventilation: a multicenter, single-blind, randomized, noninferiority trial. Crit Care Med. (2023) 51:1318–27. doi: 10.1097/CCM.0000000000005920, 37272947 PMC10497206

[21] Lu M Liu J Wu X Zhang Z. Ciprofol: a novel alternative to Propofol in clinical intravenous anesthesia? Biomed Res Int. (2023) 2023:7443226. doi: 10.1155/2023/7443226, 36714027 PMC9879693

[22] Hu J Gu X Zhu W Zhu X Ji F Luo Y . Comparison of anesthetic effects of different doses of alfentanil combined with ciprofol in elderly patients undergoing ERCP: a randomized controlled trial. BMC Anesthesiol. (2023) 23:353. doi: 10.1186/s12871-023-02325-4, 37907835 PMC10617131

[23] Wu X Liao M Lin X Hu J Zhao T Sun H. Effective doses of ciprofol combined with alfentanil in inhibiting responses to gastroscope insertion, a prospective, single-arm, single-center study. BMC Anesthesiol. (2024) 24:2. doi: 10.1186/s12871-023-02387-4, 38166724 PMC10759617

[24] Hu C Ou X Teng Y Shu S Wang Y Zhu X . Sedation effects produced by a Ciprofol initial infusion or bolus dose followed by continuous maintenance infusion in healthy subjects: a phase 1 trial. Adv Ther. (2021) 38:5484–500. doi: 10.1007/s12325-021-01914-4, 34559359 PMC8523013

[25] Teng Y Ou M Wang X Zhang W Liu X Liang Y . Efficacy and safety of ciprofol for the sedation/anesthesia in patients undergoing colonoscopy: phase IIa and IIb multi-center clinical trials. Eur J Pharm Sci. (2021) 164:105904. doi: 10.1016/j.ejps.2021.105904, 34116176

[26] Raftery S Sherry E. Total intravenous anaesthesia with propofol and alfentanil protects against postoperative nausea and vomiting. Can J Anaesth. (1992) 39:37–40. doi: 10.1007/BF03008670, 1531118

[27] Langevin S Lessard MR Trépanier CA Baribault JP. Alfentanil causes less postoperative nausea and vomiting than equipotent doses of fentanyl or sufentanil in outpatients. Anesthesiology. (1999) 91:1666–73. doi: 10.1097/00000542-199912000-00019, 10598609

[28] Xin Y Chu T Wang J Xu A. Sedative effect of remimazolam combined with alfentanil in colonoscopic polypectomy: a prospective, randomized, controlled clinical trial. BMC Anesthesiol. (2022) 22:262. doi: 10.1186/s12871-022-01805-3, 35974309 PMC9380378

[29] Rosow CE. Anesthetic drug interaction: an overview. J Clin Anesth. (1997) 9:27s–32s. doi: 10.1016/S0952-8180(97)00124-4, 9278852

[30] Pavlin DJ Coda B Shen DD Tschanz J Nguyen Q Schaffer R . Effects of combining propofol and alfentanil on ventilation, analgesia, sedation, and emesis in human volunteers. Anesthesiology. (1996) 84:23–37. doi: 10.1097/00000542-199601000-00004, 8572340

